# Immune Response and Anti-Microbial Peptides Expression in Malpighian Tubules of *Drosophila melanogaster* Is under Developmental Regulation

**DOI:** 10.1371/journal.pone.0040714

**Published:** 2012-07-12

**Authors:** Madhu G. Tapadia, Puja Verma

**Affiliations:** Cytogenetics Laboratory, Department of Zoology, Banaras Hindu University, Varanasi, Índia; University of Dayton, United States of America

## Abstract

Malpighian tubules (MT) of *Drosophila melanogaster* are osmoregulatory organs that maintain the ionic balance and remove toxic substances from the body. Additionally they act as autonomous immune sensing organs, which secrete antimicrobial peptides in response to invading microbial pathogens. We show that the antimicrobial peptides (AMP) *diptericin*, *cecropinA*, *drosocin* and *attacinA* are constitutively expressed and are regulated in developmental stage specific manner. Their developmental expression begins from 3^rd^ instar larval stage and an immune challenge increases the expression several folds. Spatial variatons in the level of expression along the MT tissue are observed. The mortality of 3^rd^ instar larvae fed on bacterial food is much less than that of the earlier larval stages, coinciding with the onset of innate immunity response in MT. Ectopic expression of AMP imparts better resistance to infection while, loss of function of one of the AMP through directed RNAi reduces host survival after immune challenge. The AMP secreted from the MT exhibit bactericidal activity. Expression of the NF-κB transcription factor, Relish, also coincides with activation of immune responsive genes in MT, demonstrating that immune regulation in MT is under developmental control and is governed by the Imd pathway.

## Introduction

Innate immunity is an evolutionarily conserved mechanism in eukaryotes and is the first line of defense which enables the organism to survive different infectious pathogens in a non-specific manner. Insects rely solely on innate immunity, which is manifested in three ways, first, a humoral response that generates circulating AMP, second, a cellular response resulting in phagocytosis or encapsulation of the intruder and third, a phenoloxidase pathway that deposits black melanin around the wounds and foreign particles [1]–[3]. Higher organisms are additionally endowed with adaptive immunity, which, opposed to innate immunity possess memory and has specificity. In *Drosophila*, the innate immunity comprises of activation of humoral response resulting in the production of AMP [4], activation and phagocytosis of pathogens by blood cells, plasmatocytes [5] and melanization by the activation of phenoloxidase pathway [2]–[3], [6]–[8].

The 20 AMP characterized in *Drosophila* can be arranged into seven different groups, viz., *cecropin*, *diptericin*, *attacin*, *drosocin*, *defensin*, *drosomycin* and *metchnikowin,* with distinct but some overlapping specificities [9], [10]–[11]. *Attacin*, *diptericin*, *cecropin* and *drosocin* are active against Gram negative bacteria, *metchnikowin* and *defensin* act against Gram positive bacteria and fungi whereas *drosomycin* is active only against fungi [12]. *Cecropin* is also induced by some of the Gram positive bacteria and fungi [13]. So far, *cecropin* has been known to have four transcripts (A1, A2, B and C), *diptericin* has two (A and B) and *attacin* has four (A, B, C and D). The expression of genes encoding AMP is under the control of Toll and Imd signaling pathways, which activate NF-κB family members. The Toll pathway is activated predominantly by the fungal and Gram positive bacteria which activates transcription factor Dorsal and Dorsal related immunity factor (Dif) and the Gram negative bacteria trigger the Imd pathway that activates NF-κB homologue transcription factor, Relish [14]–[15]. Significantly, the components of Toll and Imd pathways have orthologs in mammals, like the Interleukin1 and TNF pathway, suggesting that these pathways are evolutionary conserved and must be present in the common ancestors of invertebrates and vertebrates [16]. Mammals have also been shown to produce antibacterial defensins and cathelicidins and antifungal histatins, [17]–[18] when faced with microbial challenge.

*Drosophila* fat bodies are the functional equivalent of mammalian liver and have been implicated as the major organ, responding to systemic invasion, by secreting AMP in the hemolymph [14], [19]. However, epithelial barriers such as epithelial cells of trachea, gut, genital tract and MT act as the first line of defense and produce local response to infections [20]. Epithelial tissues provide the initial clue of impending danger because, as pathogens breach this barrier to enter into the body, they stimulate cellular and humoral defenses in the host organism. The AMP also help in maintaining a steady state of natural microflora in the system for proper functioning [17], [21]–[23]. The epithelial tissues in *Drosophila* essentially produce four different AMP, *diptericin*, *cecropin*, *drosocin* and *attacin,* regulated by the Imd pathway and with each tissue expressing at least one AMP [20], [23]–[24]. Despite the progress in the area of immune response of *Drosophila,* regulation of the tissue specific expression of AMP in barrier epithelia is still to be understood [11].

The MT of *Drosophila,* which are free floating in the hemolymph and function as osmoregulatory and detoxification organs, are now being recognized as immune sensing organ with an important role in innate immunity [25]. They can sense threat and mount effective killing response by secreting AMP, independent of fat body, the primary immune organ. The two pairs of MT are divided into initial segment, transitional segment, main segment, lower tubule and an upper and lower ureter. An earlier study [26] had identified ‘tiny cells’ apart from the two main cell types, Type I or principal cells (PC), Type II or stellate cells (SC). The tiny cells were thought to be neuroendocrine cells monitoring fluid collection [26]. However, recent studies have identified, apart from PC and SC, three other cell types based on their nuclear sizes, small, intermediate and large oval nuclei [27]. The small cells have been identified as pluripotent stem cells capable of generating all cell types of MT [27]. The principal cells are ectodermal in origin, whereas SC are mesenchymal, which undergo mesenchymal to epithelial transition and integrate in the MT during development [28]. One of the unique features of *Drosophila* MT, is the fact that they do not undergo ecdysone induced metamorphosis and are carried over from larva to adult [29]–[30], inspite of expression of proapoptotic proteins like, Reaper, Hid, Grim, Dronc and Drice [31]. Excised tubules are capable of autonomously detecting and eliminating an immune insult and the adult MT are known to express major Imd pathway associated genes like, *dredd, rel, key, imd* and *pgrp-lc* [25].

In this paper, we have studied the developmental regulation of AMP genes in MT under normal and challenged conditions, and their role in imparting resistance to *Drosophila* against pathogenic infection. We show that the entire Imd pathway associated AMP, *diptericin* ***,*** *attacinA, cecropinA* and *drosocin* are constitutively expressed in the MT and are developmentally regulated. Their expression commences from late 3^rd^ instar larval stage (110–115hrs) and persists in adults, although temporal and spatial differences in the pattern of expression of different AMP are observed and accordingly, 3^rd^ instar larvae survive the pathogenic invasion better than the 1^st^ and 2^nd^ instar larvae. We also show that the AMP produced by MT have pathogen killing potential. Over-expression of AMP in the MT of adult flies imparts better resistance to pathogens while, RNAi induced down regulation of *diptericin* makes larvae and adults sensitive to pathogens. Expression of the NF-κB homologue, Relish, begins from 3^rd^ instar larval stage, which coincides with the developmental onset of AMP expression.

## Materials and Methods

### *Drosophila* Stocks and Culture

*OregonR^+^*, *diap2 (7C)*, *cecropinA-LacZ* (kind gift from Dr. Bruno-Lemaitre), *imd* mutant, *UAS-dptB_RNAi_* (Bloomington stock centre), *diptericin-LacZ*, *diptericin-GFP, attacinA-GFP, drosocin-GFP* (kind gift from Dr. Jean-Luc Imler), *UAS-cecropinA*, *UAS-attacinA*, and *UAS-drosocin* (kind gift from Dr. Jeremy Herren), principal cell specific GAL4 driver, *c42* (kind gift by Dr. J. A. T. Dow). Flies were reared at 24±1°C on standard food containing maize powder, agar, dried yeast and sugar.

### *E. coli* Killing Assay

The *E. coli* killing assay was adopted from MCGettigan et.al., [25]. Intact MT of different developmental stages from *Oregon R* ^+^ were dissected out in Schneider’s medium and were incubated with *E. coli* for 5 hours for inducing the immune response. After 5 hours, 10 µl of medium was spotted on the marked area of bacterial lawn prepared from precultured *E. coli*. and left overnight at 37°C. Plaque formation indicated antimicrobial activity.

### *Lac-Z* Reporter Assay

MT from different developmental stages of the *LacZ* reporter stocks were dissected in Schneider’s medium. To generate immune response, MT were incubated in Lipopolysaccaharide (LPS, Sigma Aldrich) to a final concentration of 0.02 µg/ml for 3 hours. Parallel controls were maintained in Schneider’s medium without LPS. Control as well as LPS treated MT were then washed with prestaining buffer and fixed in 4% paraformaldedhyde (PFA) for 20 min., rinsed with prestaining buffer, and incubated in the staining solution (prestaining buffer plus 5 mM K3[Fe(CN)6], 5 mM K4[Fe(CN)6], 0.25% X-gal for 5 hours at 37°C. MT were mounted in 80% glycerol and observed under Nikon E-800 microscope.

### *GFP*-reporter Assay

MT of different developmental stages from the *GFP* reporter stocks were dissected out and immune challenged as for *Lac-Z* reporter stocks and MTs were fixed in 4% PFA, rinsed with PBST (1× PBS, 0.1% Triton X-100) and nuclei were stained with DAPI followed by washing with PBST. Tissues were then mounted in anti-fadent, DABCO (Sigma Aldrich). The preparations were examined on a Ziess LSM 510 Meta Confocal microscope and images were assembled using Adobe Photoshop.

### Semi-quantitative RT-PCR

Expression levels of *diptericin, cecropinA, attacinA*, and *drosocin* were determined by reverse transcriptase PCR (RT-PCR). MT from wild type 1^st^, 2^nd^, 3^rd^ instar larvae, prepupae, pupae and adult were dissected, poly (A) RNA extracted (Trizol method) and reverse transcribed with Super- script Plus (Invitrogen, USA). PCR cycle conditions were as follows: 94°C (1 min), 29 cycles of {94°C (30s), 57°C, 59°C, 60°C, (30s each), 72°C (4 min). Sequences for PCR primers for *cecropinA, diptericin* and *drosocin* were as described in Dimarcq et.al., [32], Primers for *attacinA* and *glyceraldehyde-3-phosphate dehydrogenase* (*GPDH*) used were as follows:

**Genes Primer sequence (5′-3′forward/reverse).**

*GPDH* CCACTGCCGAGGAGGTCAACTA.

GCTCAGGGTGATTGCGTATGCA.

*attacinA* GATGGACGTGCTAATCTCTG.

GGCTTAGCCGAAATGATGAG.

### *In vivo* Infection and Survival Assay

*In vivo* infection with *E. coli*, and *Mycobacterium smegmatis* (kind gift from Dr. B. N. Singh, CDRI, India) was performed by adding bacteria from the exponential log phase (3.5×10^10^ cells/ml) to fly food. Larvae of different genotypes were fed on bacterial food for 15 hours. Larvae were then transferred to normal food and numbers of surviving adult flies were counted. To confirm the ingestion of bacteria by larvae, fluorescent Alexaflour 488 *E. coli* k12 strain (Invitrogen, USA) was mixed with standard fly food for feeding. Different stages of larvae were fed for 15 hours and analyzed under Ziess LSM 510 Meta Confocal microscope.

For *in vivo* infection of adult flies, filter paper soaked in sucrose solution and containing bacteria was placed in the bottom of vial. 2–3 day old flies from wild type, *c42, UAS-cecropinA, UAS-attacin, UAS-drosocin, c42>UAS-cecropinA, c42>UAS-attacinA* and *c42>UAS-drosocin, c42>UAS-diptericin_RNAi_* were starved for 1 hour before transfer for feeding on bacteria. The numbers of dead flies in each vial were counted each day while the surviving flies were transferred to fresh vial containing filter paper soaked with sucrose solution to avoid contamination from dead flies and insufficient sucrose [33]. Each experiment was done in five replicates. The data was pooled and analyzed statistically with one way ANOVA followed by posthoc and Dunnet-t test at 0.05 level of significance.

### Immunostaining

Immunostaining with anti-Relish (1∶10, DSHB, USA) was performed as previously described in Gautam and Tapadia [34] The primary antibody was detected using Alexa Fluor488 secondary antibody (Molecular probes, USA).

## Results

### Expression of Antimicrobial Peptides in MT Commences from 3^rd^ Instar Larval Stage and Continues through Adult Stage

**Figure 1:**
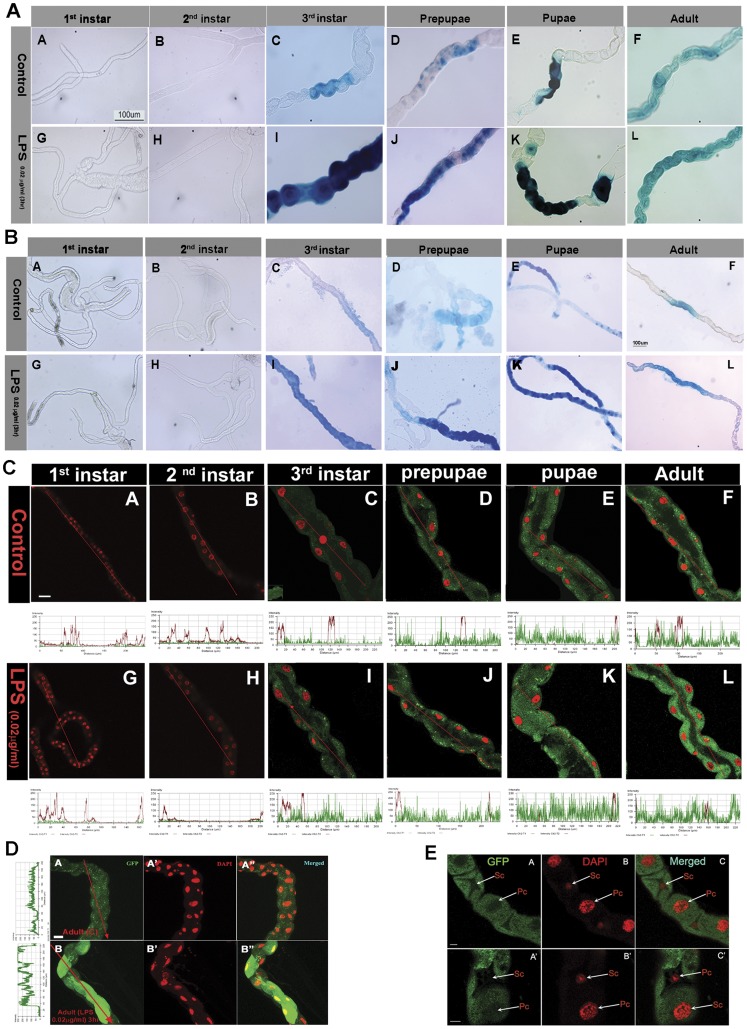
A. Expression of *diptericin* in MT at different developmental stages before and after LPS challenge. β-galactosidase staining revealed that *diptericin* does not express in 1^st^ (A) and 2^nd^ (B) instar larva under normal conditions and also after LPS treatment in 1^st^ (G) and 2^nd^ (H) instar larva. In 3^rd^ instar larva (C), pre-pupa (D), pupa (E) and adult (F) *diptericin* expression is observed under normal conditions and enhanced expression is observed after LPS treatment in 3^rd^ instar larva (I), pre-pupa (J), pupa (K) and adult (L). Scale bar represents 100 µm. **B. Expression of** ***cecropinA*** **in MT at different developmental stages before and after LPS challenge.** By performing β-galactosidase staining it was observed that *cecropinA* does not express in 1^st^ instar (A) and 2^nd^ instar (B) under normal conditions and also after LPS treatment in 1^st^ instar (G) and 2^nd^ instar (H). In 3^rd^ instar (C), pre-pupae (D), pupae (E) and adult (F) *cecropinA* expression is observed under normal conditions and enhanced expression is observed after LPS treatment in 3^rd^ instar (I), pre-pupae (J), pupae (K) and adult (L). Scale bar represents 100 µm. **C. Expression of** ***drosocin*** **in MT at different developmental stages before and after LPS challenge.** Green fluorescence of GFP-reporter in *drosocin*-promoter construct shows that *drosocin* does not express in 1^st^ (A) and 2^nd^ instar larva (B) under normal conditions and also after LPS treatment in 1^st^ (G) and 2^nd^ (H) instar larva. In 3^rd^ instar larva (C), pre-pupa (D), pupa (E) and adult (F) *drosocin* expression is observed under normal conditions and expression enhanced after LPS treatment in 3^rd^ instar larva (I), pre-pupa (J), pupa (K) and adult (L). Graph below each panel shows the line profile display made in LSM 510 meta confocal microscope to measure fluorescence intensity (Red arrow indicates the region used for the measure). The increase in intensity after LPS treatment is highest in adult and then pupa, prepupa and 3^rd^ instar. The nuclei were stained with DAPI (pseudocolour red). Scale bar represents 20 µm. **D. Expression of** ***attacin A*** **in adult MT.** Green fluorescence of GFP-reporter in *attacinA*-promoter construct shows that *AttacinA* expresses only in the adult MT before (A) and after (B) LPS challenge. Nuclei were stained with DAPI before (A’) and after (B’) LPS treatment. Graph on the left was created using line profile display of LSM 510 meta confocal microscope to measure the intensity of fluorescence which showed that the intensity of GFP was much more in LPS treated MT than without LPS (Red arrow indicates the region used for the measure). The nuclei were stained with DAPI (pseudocolour red). Scale bar represents 20 µm. **E**. **Expression of** ***diptericin*** **in principal cells of MT.** Green fluorescence of GFP shows that diptericin expresses in PC of MT. No GFP signal was observed in SC (A). Nuclei were counterstained with DAPI (B) which distinguishes PC and SC based on their nuclear size. Merged images show PC containing large DAPI stained nucleus with GFP signal and SC with small DAPI stained nucleus with no GFP signal (C). Higher magnification shows clear view of PC and SC (A’, B’, C’). PC  =  Principal cells, SC  =  Stellate cells. Scale bar represents 20 µm.

We have examined expression of four AMP, *diptericin, cecropinA, attacinA* and *drosocin* induced by Gram negative bacteria, and which are activated by the Imd pathway. Taking advantage of a robust survival and physiological activity of MT in culture medium [35]–[36] and their capability to respond to LPS challenge [25], we monitored the β-galactosidase activity of *Lac-Z* reporters under *diptericin* and *cecropinA* promoters and green fluorescence in GFP construct under *drosocin* and *attacinA* promoters after exposing the isolated MT to LPS challenge. The *diptericin* (Figures 1AA and 1AB), *cecropinA* (Figures 1BA and 1BB), *drosocin* (Figures 1CA and 1CB) and *attacinA* (not shown) are not expressed in 1^st^ and 2^nd^ instar larval MT under unchallenged condition. To find out whether the AMP in these larvae are expressed after immune challenge, we subjected the 1^st^ and 2^nd^ instar MT to LPS treatment (Section 2.3), however, we did not find any expression of *diptericin* (Figures 1AG and 1AH), *cecropinA* (Figures 1BG and 1BH), *drosocin* (Figures 1CG and 1CH) and *attacinA* (not shown) even after the LPS challenge. These results showed that, unlike the inducible expression of *cecropinA1* in the fat bodies of 1^st^ and 2^nd^ instar larvae after immune challenge [21] the AMP genes in early developmental stages in MT are refractory to immune challenge.

Expression of *diptericin* (Figure 1AC), *cecropinA* (Figure 1BC) and *drosocin* (Figure 1CC) was first observed in 3^rd^ instar larvae (110–115 hrs) and thereafter expression of *diptericin* (Figures 1AD, E and F), *cecropinA* (Figures 1BD, E and F) and *drosocin* (Figures 1CD, E and F) was observed in prepupal, pupal as well as adult stages. After *in vitro* immune challenge with LPS, enhanced expression of all the three AMP was observed, though the response was not same for all the AMP. The strongest response was observed for *diptericin* after LPS treatment and the expression was stronger in larva (Figures 1AI), prepupa (Figures 1AJ) and pupa (Figures 1AK) than in adult MT (Figures 1AL). *cecropinA* expression increased only moderately after LPS treatment in 3^rd^ instar larva (Figures 1BI), prepupa (Figures 1BJ), pupa (Figures 1BK) and adult (Figures 1BL). *Drosocin* expression after LPS treatment in pupa (Figures 1CK) and adult (Figures 1CL) was greater than in 3^rd^ instar larva (Figures 1CI) and prepupa (Figures 1CJ). The increase in GFP expression was quantified by line profile display function of LSM meta 510 confocal microscope (shown below the image). *AttacinA* expression was observed only in the adult MT (Figure 1DA) and the expression increased after immune challenge (Figure 1DB). The increase in *attacinA-*GFP was again quantified by the line profile display (shown adjacent to the image), which showed a significant increase in fluorescence after immune challenge (Figures 1DA and B). Thus while all the AMP respond to LPS treatment, the extent of response is in a developmental manner. The expression pattern was also not uniform throughout the tubule. Using *diptericin-GFP* we observed that *diptericin* expresses only in the SC but not in the PC (Figure 1EA, Figure 1EA’), DAPI (Figure 1EB, Figure 1EB’), staining clearly differentiates between larger PC and smaller SC. The merged images (Figure 1EC, C’) clearly show that there is no expression of *diptericin* in SC. This is in agreement with the earlier report [25]. On the other hand, *drosocin* (Figure 1C), *attacinA* (Figure 1D) and *cecropinA* (not shown) expresses in both, PC and SC of MT. Thus we conclude that the Imd pathway regulated AMP express in the MT in a developmental stage specific manner but they do not follow all or none rule.

**Figure 2:**
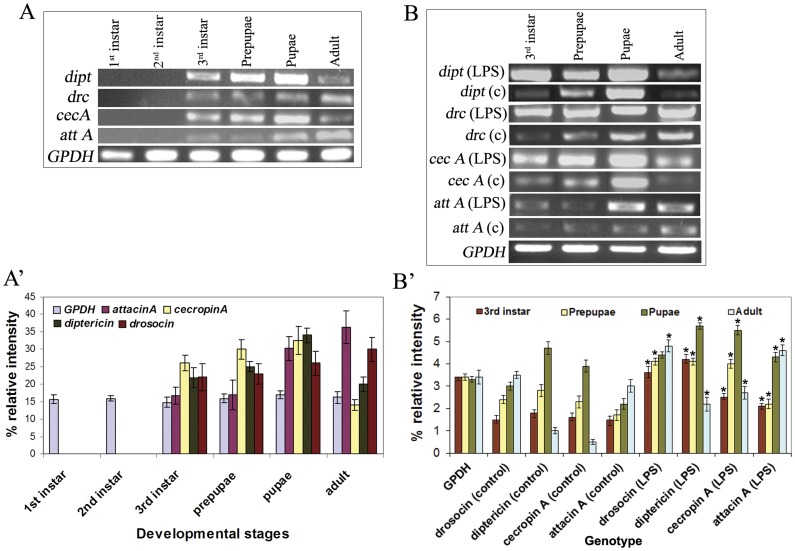
RT-PCR for detecting AMP gene transcripts during development and after LPS treatment. Expression of, *Diptericin (dipt), drosocin (drc), cecropinA (cecA) and attacinA (attA),* in MT from 1^st^, 2^nd^, 3^rd^, prepupae, pupae and adult under normal conditions (A). The intensity of bands were measured and plotted on a graph (A’). *AttacinA* and *Drosocin* is highest in adult, whereas *diptericin* and *cecropinA* is highest in pupae. MT from 3^rd^ instar, prepupae, pupae and adult were treated with LPS and RT-PCR was done (B). Enhanced expression of all the AMP were observed after LPS treatment (LPS) and compared with control (C) without LPS treatment. The intensity of bands were measured and plotted on a graph (B’) which show that after immune challenge there is an enhanced expression of all AMP at all stages of development. *Glyceraldehyde-3-phosphate dehydrogenase (GPDH)* is used as an internal control to ensure the integrity of RT-PCR.

The reporter gene expressions were further substantiated by semi quantitative RT-PCR analysis with and without LPS challenge (Figure 2). Expression of *diptericin, cecropinA,* and *drosocin* was first observed from 3^rd^ instar and there was no expression of any of these in 1^st^ and 2^nd^ instar MT which correlated very well with reporter gene assay. However, although *attacinA* GFP expression was observed only in the adult under normal conditions, RT-PCR results showed presence of *attacinA* transcripts in 3^rd^ instar larvae as well. This discrepancy could be because of the low level of expression (Figures 2A and 2A’) or because of less sensitivity of the GFP assay than RT-PCR or because of posttranscriptional control mechanism. In the later developmental stages, prepupae, pupae and adult, transcripts of all the AMP including *attacinA* were seen (Figure 2A). Results showed that *diptericin* and *cecropinA* expression was highest at pupal stages which declined at adult stage while *attacinA* and *drosocin* expression increased gradually from 3^rd^ instar larval stage with maximum in adults (Figures 2A and 2A’). RT-PCR was also carried out with RNA from MT after LPS challenge (Figure 2B) which showed significant induction of all the AMP (Figures 2B and 2B’) suggesting that the ability to respond to immune challenge does not diminish at any developmental stage. The sizes of all the transcripts were as expected.

### MT can Mount Killing Activity Independent of fat Bodies and Hemolymph

**Figure 3:**
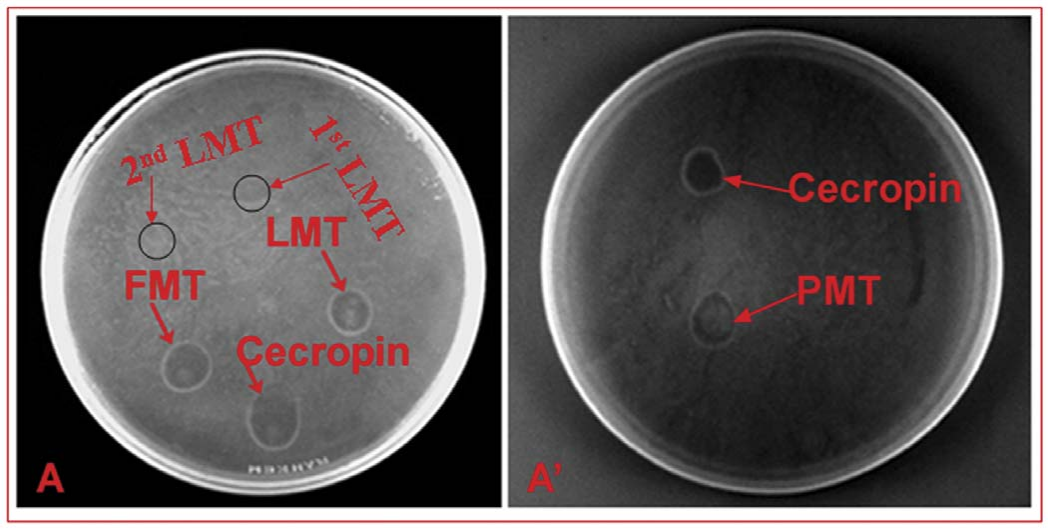
*E. coli* killing assay. The plates have *E. coli* bacterial lawn grown upon them. No plaque was formed when 1^st^ instar **(**A, 1^st^ LMT) and 2^nd^ instar (A, 2^nd^ LMT) exudate from MT were plated on bacterial lawn (encircled region). Plaque was observed when exudate from wandering 3^rd^ instar larval MT (A, LMT), pupal MT (B, PMT) and adult MT (A, FMT) were plated on bacterial lawn. *cecropin* was used as +ve control which resulted in the formation of plaque.

To examine whether the AMP produced in 3^rd^ instar larval stage correlated with antimicrobial activity, we performed the *E. coli* killing assay. The MT were incubated with *E. coli* to stimulate AMP production and the exudate was added to a bacterial lawn. The appearance of plaques was indicative of bactericidal activity. Plaques were observed on bacterial lawn when extracts from 110–115 hrs 3^rd^ instar larvae (Figure 3A LMT), pupae (Figure 3A’ PMT) and adult (Figure 3A FMT) were used. No plaque formation was observed with exudates from 1^st^ and 2^nd^ instar larval MT (Figure 3A 1^st^ LMT, 2^nd^ LMT). To confirm that the plaques were actually a result of killing *E. coli* cells, we used synthetically available antimicrobial peptide, *cecropin*, as a positive control. The morphology of plaque formed by spotting *cecropin* (Figure 3A’, *cecropin*) was the same as formed by MT, confirming that plaques formed were actually due to the killing of *E. coli.* These results showed that the MT from 3^rd^ instar larval stage have autonomous immune competence and thus are able to mount killing activity independent of fat bodies and hemolymph.

### 3^rd^ Instar Larvae are More Resistance to Infection than 1^st^ or 2^nd^ Instar

**Figure 4:**
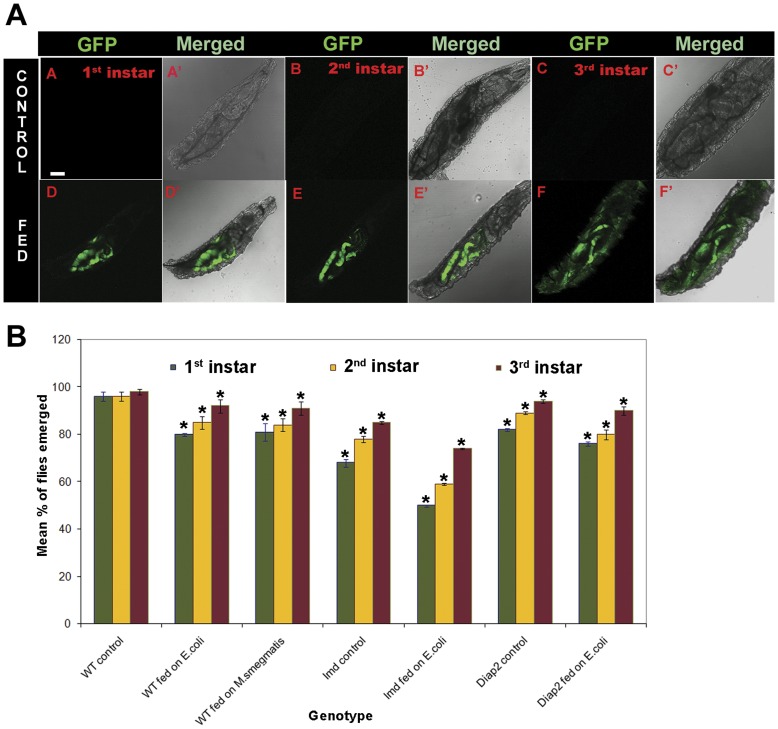
A. *E. coli-GFP* expression in gut of larvae. No fluorescence is observed in the gut of control larvae, not fed on GFP bacteria of 1^st^ instar (A) 2^nd^ instar (B) and 3^rd^ instar (D). A’, B’ and C’ are DIC images merged with fluorescence shows that the gut region is devoid of GFP-bacteria. Green fluorescent is observed when 1^st^ (D), 2^nd^ (E) and 3^rd^ (F) instar larvae are fed on GFP-bacteria. DIC images and merged fluorescence (D’, E’ and F’) confirms that green fluorescence is in the gut. **B**. **Mortality rate of different stage larvae when fed on bacterial food.** Percentage of adults emerged shows that 1^st^ instar (green graph) wild type larvae are most susceptible to *E. coli* and *M. smegmatis* than 2^nd^ instar (yellow graph) and 3^rd^ instar (maroon graph). *imd* mutants (control) are less viable than *diap2* mutants (control) and also the mortality rate for *imd* mutants are significantly less than *diap2* mutant when fed on *E. coli.* Asterick (*) represents significance at p<0.05.

Since our results showed that AMP expression commences from 3^rd^ instar larval stage and the AMP produced can mount effective killing activity, we wanted to check the survival of wild type larvae when fed on Gram negative *E. coli* and Gram positive *M. smegmatis* bacteria. To confirm that larvae consume bacteria mixed with food, we used fluorescent *E. coli* (Invitrogen, USA) to feed larvae. Different stages of larvae were fed on bacteria mixed food, and green fluorescence was observed in 1^st^, 2^nd^ and 3^rd^ instar larvae (Figures 4AD, E and F) indicating the presence of the fed bacteria in gut. As expected the unfed control in 1^st^, 2^nd^ and 3^rd^ instar larvae (Figures 4AA, B and C) did not show fluorescence. DIC images of the same larvae (Figures 4AA’ B’, C’, D’ E’ and F’) confirmed the presence of GFP in the gut region. Wild type larvae from different developmental stages were fed on food containing *E.coli* or *M. smegmatis*, for 15 hrs following which they were transferred to normal food and the percentages of adult survivors were calculated (Figure 4B). Compared to the *E. coli* fed 3^rd^ instar larvae and the unfed controls, a significantly greater pre-adult lethality was observed when 1^st^ or 2^nd^ instar larvae were fed on *E*. *coli*. A similar trend was observed after feeding on *M. smegmatis* (Figure 4B). These results confirmed that the 1^st^ and 2^nd^ instar larvae were more susceptible to pathogenic insults compared to 3^rd^ instar, as expected from our above finding that the MT and other immune tissues are not immune-competent during early larval stages. A very high lethality was not observed because of the other innate immunity protective mechanisms and expression of *cecropin* in early 1^st^ instar [23]. Our results also suggest that the induced immune response does not differentiate between Gram negative and Gram positive bacteria. We also carried out the survival assay on two Imd pathway mutants, *imd* and *diap2*. Since the Imd pathway is predominantly activated by Gram negative bacteria [12], [37], we subjected these mutants to feeding on *E. coli*. Comparison of survival of these mutants with wild type grown on normal food showed that viability of *imd* and *diap2* mutants is significantly less compared to wild type (Figure 4B) since only 50% of the *imd* and 76% of *diap2* mutant adult flies emerged when 1^st^ instar larvae were fed. Similarly when 2^nd^ instar larvae were fed only 59% of *imd* and 80% of *diap2* mutant flies emerged. The number of surviving adults was highest when 3^rd^ instar larvae were fed on *E. coli* containing food. The survival curve shows that in the mutants too, the ability to fight infection increases with age, although compared to wild type the response was significantly less. The *imd* mutant was more sensitive to the pathogenic infection than the *diap2,* which could be because Imd has a critical role in transducing the signal from the cell exterior to the nucleus. As shown earlier [38], and confirmed by present results it can be stated that immune sensing by epithelial tissues is critical to survival of the organism.

### Over-expression of AMP in MT Enhance the Ability of Adult Flies to Fight Infection

**Figure 5:**
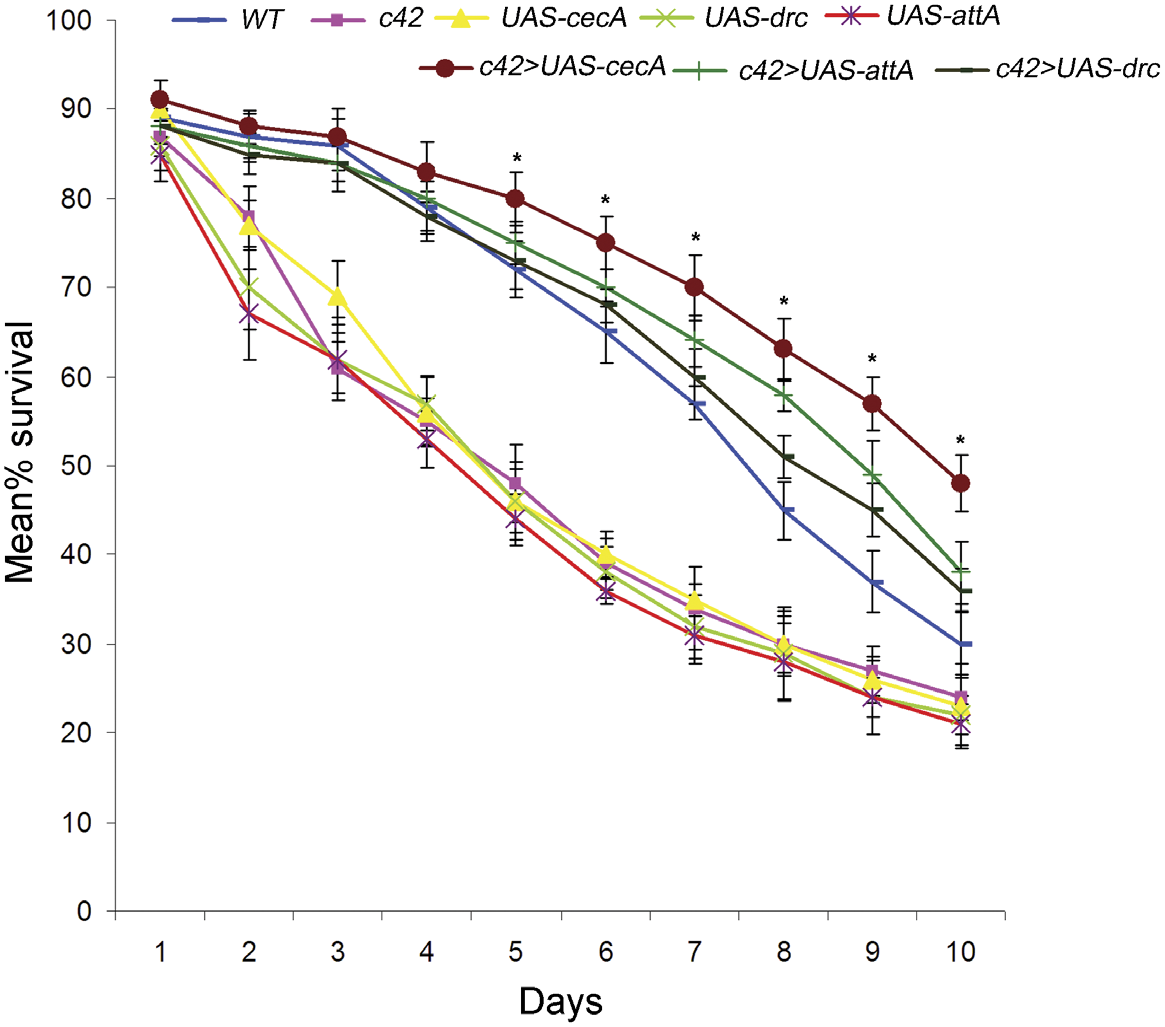
Survival rate following bacterial infection increases after over expressing AMP in MT of adult flies. Percentage survival of *c42>UAS-cecropinA* (maroon graph), *c42>UAS-attacinA* (green graph) and *c42>UAS-drosocin* (black graph) flies are significantly more in comparison to wild type (blue graph), *c42* (pink graph ), *UAS-cecropinA* (yellow graph), *UAS-attacinA* (red graph) *UAS-drosocin* (olive green graph) flies when fed on *E. coli*. Asterick (*) represents level of significance at p<0.05.

Since the above results showed that MT express AMP in developmental stage specific manner, which also coincides with the ability to fight infection, we next wanted to find the importance and role of MT in resisting infection. For this we directed over expression of specific AMP in MT and examined the effect on survival of flies fed on pathogens. The number of live flies reduced continuously and by day 10 only 30% of the flies were alive (Figure 5). In another set, we over expressed each of the AMP individually (*cecropinA*, *attacinA* or *drosocin*) using the *UAS-Gal4* system [39] in the MT using *c42 Gal4* driver and *UAS-cecropinA*, *UAS-attacinA* or *UAS-drosocin.* To rule out a possible effect of the transgene in the viability, each of these stocks individually were also subjected to the viability assay. Flies in the undriven transgene stocks also displayed death of flies which though appeared greater than that observed in wild type, the difference was not statistically significant (Figure 5). Therefore, the transgenes by themselves did not confer any advantage to the flies. Feeding the *c42>UAS-cecropinA, c42>UAS-attacinA* and *c42>UAS-drosocin* flies on pathogenic food revealed an increase in percentage survival in each case when compared to wild type. Maximum rescue was observed in *UAS-cecropinA* expressing flies followed by *UAS-attacin* and least with *UAS-drosocin* suggesting that the AMP expression in MT conferred a definite advantage to the flies. However, these data also suggested that the different AMP do not confer similar immunity with *cecropinA* appearing to be the most potent AMP compared to *drosocin* and *attacinA*. This may also explain the low level of *cecropin* observed in unchallenged and challenged conditions (Section 3.1).

### Depletion of *diptericin* in MT Reduces the Ability of 3^rd^ Instar Larvae and Adult Flies to Survive Infection

**Figure 6:**
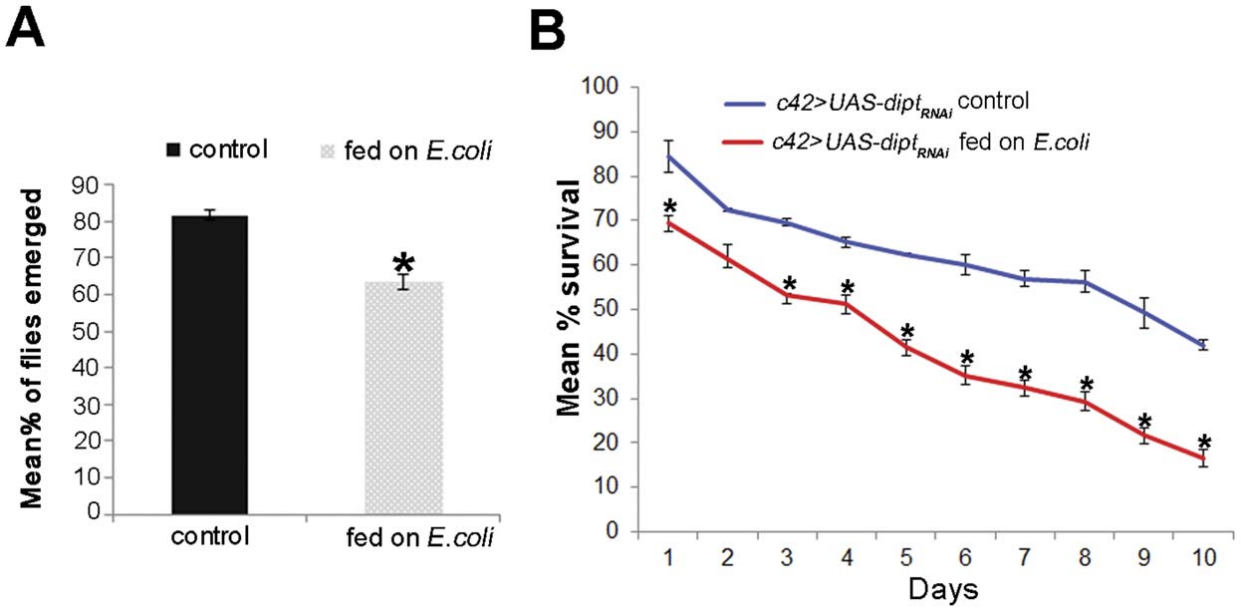
A. Mortality rate of 3^rd^ instar larvae increases after depleting diptericin in MT. Percentage of adult flies eclosed from 3^rd^ instar larvae of *c42>UAS-diptericin_RNAi_* fed on *E. coli* is decreased (dotted graph) in comparison to control *c42*>*UAS-diptericin_RNAi_*3^rd^ instar larvae (black solid graph). Asterick (*) represents level of significance at p<0.05. **B. Survival rate following bacterial infection decreases after depleting** ***diptericin*** **in MT of adult flies**. Percentage survival of *c42*>*UAS-diptericin_RNAi_* flies fed on *E. coli* (red graph) significantly reduces in comparison to *c42>UAS-diptericin_RNAi_* control unfed flies (blue graph). Asterick (*) represents level of significance at p<0.05.

As over-expression of AMP (*Cecropin, drosocin and attacinA*) in MT result in enhanced immune protection, we examined the survival when *diptericin* was down- regulated in MT using *UAS-diptericin_RNAi._* Since the expression of AMP start from 3^rd^ instar larvae, we checked the survival of 3^rd^ instar larvae expressing UAS-*diptericin_RNAi_* under the *c42* driver following 15 hrs feeding on *E. coli*. As control, larvae of the same genotype were fed on food without *E.coli*. Results presented in Figure 6A show that *E. coli* fed larvae expressing *diptericin_RNAi_* transgene in MT showed reduced survival as adults (63%) than those not fed on bacteria (82%). Statistical analysis showed the difference to be significant. We also measured the survival of adult flies expressing *diptericin_RNAi_* transgene in MT after feeding them on *E. coli*. It was observed that again there is a decrease in the survival of *c42>UAS-diptericin_RNAi._* after feeding on pathogen containing food. Only 17% of *c42>UAS-diptericin_RNAi._* flies survived after ten days of infection compared to 42% surviving *c42>UAS-diptericin_RNAi._*flies fed on control, non-pathogenic food (Figure 6B).

### Relish Expression in MT Begins at 3^rd^ Instar Larvae

**Figure 7:**
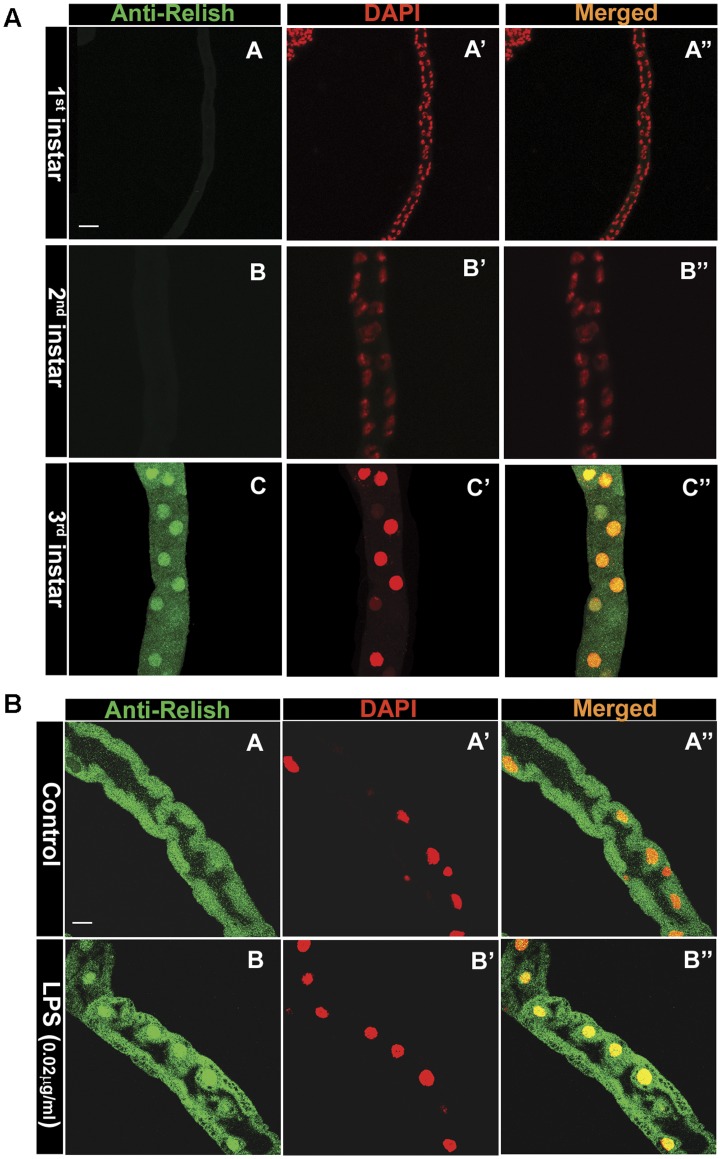
A. Expression of Relish in MT during development. Immunostaining using anti-Relish showed that in the 1^st^ (A) and 2^nd^ (B) instar larvae Relish expression is not observed but in the 3^rd^ instar (C) we do observe Relish staining. Counterstaining was done with DAPI (A’, B’ and C’) pseudo color red. Merged images (A”, B” and C”) show expression to be cytoplasmic as well as nuclear. **B.** **Relish expression after immune challenge.** Number of nuclei showing Relish expression is enhanced after LPS treatment of 3^rd^ instar larvae (B) compared to control (A). Nuclei are counter stained with DAPI (A’ and B’), pseudo color red and merged images are A” and B”.

Epithelial immune response occurs via Imd dependent pathway leading to the activation of Relish [1], [12], [40]. Relish has inhibitory ankyrin repeats at the COOH terminus which when removed by endoproteolytic cleavage, results in the release of transcriptionally active Rel Homology (RH) domain, allowing its translocation into the nucleus and binding to enhancer elements in the promoter of antimicrobial genes [41]. Localization of Relish is important for its transcriptional activity and we used this parameter to check the activation of Imd signaling in MT at different larval stages (Figure 7A). Relish expression was not observed in the 1^st^ or 2^nd^ instar MT (Figures 7AA and 7AB) which correlates with the absence of AMP expression at these stages. Relish expression was first observed in the 3^rd^ instar larval MT under unstimulated condition (Figure 7AC). Localization of Relish was predominantly cytoplasmic, though in some cells we also observed its presence in nuclei as well (Figure 7AC’ and 7AC”). Nuclear localization of Relish could be responsible for the basal levels of AMP observed in 3^rd^ instar without the immune challenge. Enhanced Relish expression was observed after LPS challenge (Figure 7BB) in 3^rd^ instar larval MT in comparison to unchallenged condition (Figure 7BA). LPS treatment also led to a greater (29%) incidence of cells showing Relish localization in the nucleus. A nuclear localization of Relish in 3^rd^ instar larval MT is suggestive of the activation of Imd signaling.

## Discussion

MT constitute an important branch of humoral immunity though their primary function in insects is to maintain fluid homeostasis. We present here an extensive analysis of the immune phenotype of the MT. One of our most significant observations is that there is a constitutive expression of all the Imd pathway regulated AMP genes, *diptericin, attacinA, cecropinA* and *drosocin* in the MT of healthy unchallenged individuals, unlike the other epithelial structures [24], and fat bodies [42]–[43], which are known to express AMP only after the immune challenge. The MT also mount an efficient immune response by enhancing the expression of *diptericin, attacinA, drosocin and cecropinA* evidenced by the bactericidal activity (observed in the present study). Increased survival following over-expression of the AMP and reduced viability following their RNAi-based down-regulation in MT of individuals challenged with bacteria further shows an important role of AMP expression in MT in the immune response.

Contrary to an earlier report that *cecropin* expression is not increased after immune challenge in the adult MT [25], our study clearly showed an enhanced expression of *cecropinA* after LPS treatment. Indeed the maximum rescue of bacteria-fed adult flies following over-expression of *cecropinA* in the MT maximally, further suggest that *cecropin* may be one of the most potent peptides against pathogens.

Although fat bodies are considered the primary immune organ of the insects [42]–[43], based on our observations we propose that the MT, by virtue of expressing AMP constitutively, provide the immediate immune protective response before the fat bodies respond to the insult and the organism develops immune competence. The importance of MT as immune organ stems from the fact that they are free floating in the hemolymph and one of the first epithelial tissues to sense systemic invasion of microbes.

The steroid hormone ecdyosne coordinates the progressive changes in post embryonic development in insects and also modulates cellular and humoral innate immunity [44]. Although there is a general consensus that during early stages of development the expression of AMP is low or absent, there are contradictory reports about their expression during later stages of development and metamorphosis. Several groups have reported a negative correlation between ecdysone and immune response [14], [23]–[24]. Low levels of *cecropinA* [45], and *diptericin* [46] have been reported in pupal fat bodies in response to pathogenic invasion [47]. Another study also reported constitutive expression of *diptericin* in very few late larva and pupae. A negative correlation between ecdysone levels and AMP expression has also been reported in *Calliphoravicina* and *Drosophila* during late pupal stages [48]. On the other hand, other reports suggest that ecdysone regulates AMP production in flies and mbn-2 cells in a positive manner [32], [49]–[50]. Together these results suggested that ecdysone impacts the expression of AMP positively as well as negatively possibly because of some other factors also being involved in the regulation. Ecdysone, however positively regulates other aspects of immunity such as activation of *Prophenoloxidase I* gene in *Anopheles* which contains ecdysone receptor elements and is enhanced by ecdysone hormone [51]–[52], and differentiation of mbn-2 cells into macrophages leading to increased phagocytic behavior [32]. It also leads to induction of hemolin expression in fat body of diapausing pupae of *Cecropia* moth [53]. Juvenile hormone on the other hand inhibits ecdysone signaling in a stage specific manner and acts as an immune-suppressor in *Drosophila* but in post-embryonic development of *Bombyx mori* juvenile hormone levels acts as an immune activator as compared to ecdysone which inhibits innate immunity [54]. Thus a delicate balance between the juvenile hormone and ecdysone regulates several pathways including the innate immunity, which greatly depends on the developmental stage and is species specific. In vertebrates too, hormones and nuclear hormone receptors regulate adaptive and innate immunity [55]–[57]. In mammals estrogen receptors, glucocorticoid receptors, vitamin D receptors and other nuclear hormone receptors have been implicated in regulating innate immunity and proinflammatory gene expression [56].

Interestingly, the present results show that MT gain immune competence at 3^rd^ instar larval stage and continue to express AMP throughout adult. The beginning of AMP expression coincides with high peak of ecdysone although, there is considerable variation in the level of expression of the different AMP. *Diptericin* and *cecropin* levels are high at pupal stage coinciding with high levels of ecdysone compared to reduced *attacinA* and *drosocin* levels. However, at no stage a complete absence of AMP production in response to high levels of ecdysone at pupation was observed. Expression of AMP during the pupal stages in MT is significant since MT are one of the tissues that do not undergo ecdysone induced destruction [31].

We suggest that MT, being not histolysed during pupal metamorphosis hold a crucial position in the innate immune response specifically during metamorphosis, when fat bodies and other AMP producing tissues are histolysed by programmed cell death [29]–[30]. Expression of AMP in the MT during pupal stages in unchallenged and challenged condition provides a safeguard to the holometabolous insects. MT are analogous to human kidney in the terms of development and function. The nephrons of vertebrate kidney originate from ectodermal and mesenchymal tissues, similar to MT whose PC originate from ectodermal lineage while SC are mesenchymal in origin [28]. PC and SC are the two developmentally and functionally distinct major cell types of MT [34], [58]. Our results show that these cells also respond differentially to immune challenge. Since while *diptericin* is secreted only by the PC, *attacinA* and *drosocin* are secreted by PC as well as SC. Similar to SC of mesenchymal origin, human mesenchymal stem cells (MSC) also secrete AMP like LL-37 against Gram-negative bacteria [59], suggesting that the response to immune challenge may be conserved in evolution. Recent immune studies indicate that MSC may have beneficial effects in the treatment of sepsis caused by bacterial infection [59]. Major disorders such as inflammatory bowel disease [60], Crohn’s disease [61], and asthma [62] are caused by deregulation of epithelial immune defense. Since epithelial cells from *Drosophila* and human share substantial similarities [63], MT appear to be highly suitable for modeling human renal diseases related to dysfunction of innate immune system [64].

### Conclusion

Epithelia tissues act as the first line of defense [21]–[22], and MT are specifically important since they are free floating in the hemolymph and are one of the first epithelial tissues to sense systemic invasion of microbes. Our study shows that MT gain immune competence at 3^rd^ instar larval stage and constitutively express *diptericin*, *cecropinA, drosocin* and *attacinA* till adult stage. The expression of Relish also coincides with the expression of AMP suggesting that the expression of AMP is transcriptionally regulated. A constitutive expression of AMP which, has bactericidal activity by the MT is important for the organism to fight infection.
