# Correction: Immune Response and Anti-Microbial Peptides Expression in Malpighian Tubules of *Drosophila melanogaster* Is under Developmental Regulation

**DOI:** 10.1371/annotation/4b02305d-dcb8-40db-8f1f-1f7f0da51544

**Published:** 2012-08-09

**Authors:** Puja Verma, Madhu G. Tapadia

There was an error in the author order in the by line. Puja Verma is the correct first author and Madhu G. Tapadia is the correct second author. 

The correct citation is: Verma P, Tapadia MG (2012) Immune Response and Anti-Microbial Peptides Expression in Malpighian Tubules of Drosophila melanogaster Is under Developmental Regulation. PLoS ONE 7(7): e40714.

